# Caffeine as an Active Molecule in Cosmetic Products for Hair Loss: Its Mechanisms of Action in the Context of Hair Physiology and Pathology

**DOI:** 10.3390/molecules30010167

**Published:** 2025-01-04

**Authors:** Ewelina Szendzielorz, Radoslaw Spiewak

**Affiliations:** Department of Experimental Dermatology and Cosmetology, Jagiellonian University Medical College, ul. Medyczna 9, 30-688 Krakow, Poland; ewelina.szendzielorz@uj.edu.pl

**Keywords:** caffeine, hair loss, alopecia, effluvium, treatment, metabolic pathways, skin penetration

## Abstract

Caffeine has recently attracted attention as a potential remedy for hair loss. In the present review, we look into the molecule’s possible mechanisms of action and pharmacodynamics. At the molecular level, it appears that the physiological effects of caffeine are mainly due to the molecule’s interaction with adenosine pathways which leads to an increase in cAMP level and the stimulation of metabolic activity in the hair follicle. Moreover, caffeine also acts as an antioxidant and may prevent degenerative processes. While the intact stratum corneum seems virtually impenetrable to caffeine and a range of physical and chemical methods have been proposed to facilitate its penetration, hair follicles seem to be both a main entry route into the skin and target structures for caffeine at the same time. Caffeine readily forms bonds with water and other molecules which may influence its bioavailability and should be taken into account when engineering future hair products. The results of clinical studies published so far seem promising; however, the majority of the studies of caffeine-based hair loss products offer a very low level of evidence due to considerable flaws in study designs. Nevertheless, the metabolic activity of caffeine and its ability to enter and accumulate in the hair follicles combined with the results of available clinical trials seem to indicate that caffeine could indeed prove as an effective and safe option in the management of hair loss.

## 1. Introduction

Over 2000 different substances have been isolated from *Coffea arabica* (“Arabica”) and *Coffea canephora* (“Robusta”)—two cultivars used for the crop production of coffee beans. The most famous among these molecules is caffeine, which amounts to 1.3–2.4% total bean weight [1]. Caffeine is known to exert a range of metabolic and pharmacological effects [2,3,4,5]. Also, in cosmetic applications, caffeine is gaining importance [6,7]. In the present article, we take a closer look at this molecule with regard to its possible use as a hair loss-preventing and hair growth-stimulating agent (Table 1).

At present, only two pharmacological agents are approved for hair loss by the FDA and EMA: topical minoxidil and oral finasteride [9]. The efficacy of these drugs is confirmed in clinical trials; however, treatment may be hampered by adverse events, unsatisfactory compliance, and a high relapse rate. Finasteride may cause decreased libido, headaches, and gastrointestinal problems [10]. Numerous cases of allergic contact dermatitis to topical minoxidil are reported throughout the medical literature [11]. Other pharmaceutical products used hair loss are not registered for hair loss treatment (off-label use) which may create legal problems in some countries with regard to reimbursement and doctor’s liability. On the other hand, the market is flooded with numerous haircare products, each promoted as an effective remedy for hair loss—a claim that in most cases lacks any scientific evidence [12,13,14]. Compared with that, caffeine seems to have a good safety profile as well as promising experimental and clinical data.

The terms “caffeine” and “coffee” are often confused in both biomedical literature and popular opinion. It is important to remember that they are not synonyms, and the biological effects of coffee cannot be reduced to the isolated effects of caffeine [15]. Caffeine is considered an active molecule in various contexts, most notably as a central nervous system (CNS) stimulant [16,17,18]. Nevertheless, it also acts directly in the peripheral tissues and cells by influencing their vital processes. The key physiological effects of caffeine in the context of the skin and its appendages include the following [19,20]:

- Phosphodiesterase inhibition—caffeine acts as a phosphodiesterase inhibitor that prevents the breakdown of cyclic adenosine monophosphate (cAMP), thus contributing to its accumulation in the cell. Elevated levels of cyclic AMP (cAMP) upregulate cell signaling pathways that promote cell proliferation and metabolic activity in the skin cells and hair follicles (HFs).
- Adenosine receptor antagonism—caffeine blocks adenosine receptors, specifically the A1 and A2A subtypes. This results in vasodilation and increased microcirculation in the skin and HFs, with increased blood flow facilitating better oxygen and nutrient delivery as well as waste removal.
- Antioxidant properties—caffeine also has antioxidant effects which makes it capable of neutralizing free radicals that could otherwise damage skin cells and HFs. Thus, caffeine counteracts oxidative stress, a factor contributing to skin aging and HF degradation (hair loss; balding).
- Stimulation of IGF-1 expression—caffeine has been demonstrated to increase the expression of insulin-like growth factor 1 (IGF-1) in HFs. IGF-1 has been implicated as a factor initiating and sustaining the anagen (growth) phase in the hair cycle, thus promoting hair growth and reducing hair loss.

The above list illustrates caffeine’s potential as an active ingredient in skin and hair care products, with both therapeutic and cosmetic applications. Therefore, the aim of the present review was to collate the published information on the role of caffeine as a molecule with the potential to combat hair loss.

This narrative review was conducted in parallel with a systematic review of clinical trials of caffeine against hair loss [21]. Between January and November 2024, a query was carried out in PubMed, Scopus, and Web of Science with the combination of search words such as “caffeine”, “1,3,7-trimethylpurine-2,6-dione”, “hair”, “alopecia”, “effluvium”, “bald”, “pilo *”, or “pili”. No language filters or publication date limits were applied. The initial search returned 1695 articles, among which 573 duplicates were identified and removed. The resultant 1122 articles were assessed by title and abstract and 71 articles that passed the initial screening underwent a full-text assessment. Further searches, carried out between October and December 2024 were aimed at specific questions regarding the properties and effects of caffeine at a molecular, cellular, and tissue level that emerged during the writing process. At this stage, PubMed and Google Scholar were used for ad hoc queries with publication date limit set as “since 2020” to yield the most recent papers. These aimed searches resulted in a total of 186 additional papers that underwent the full text analysis. Reference lists of these articles also were screened for publications of relevance to the topic with a further 17 publications of interest identified. After the analysis of content, 127 papers were finally included into the present review.

## 2. Caffeine as a Chemical Molecule

**Figure 1:**
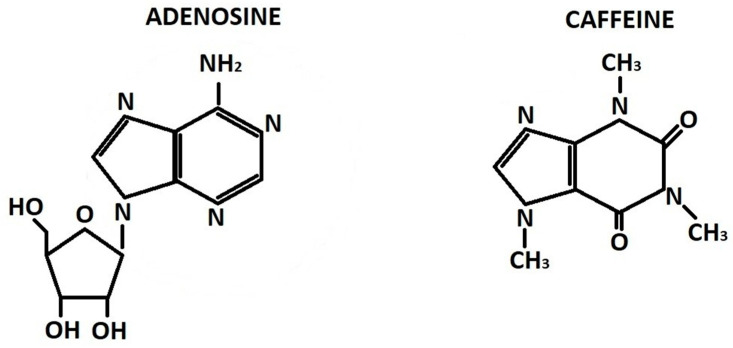
Chemical structure of adenosine (L) and caffeine (R).

Caffeine is a purine alkaloid trimethylxanthine in which three methyl groups are located at positions 1, 3, and 7 of the double purine ring (Figure 1). The molecule occurs naturally in seeds, leaves, and fruits of over 60 different plant species originating from Africa, South America, and East Asia in which it acts as a natural insecticide, herbicide, and repellent. Aside from agricultural production, caffeine may also be synthesized on an industrial scale [22]. Natural caffeine is consumed by people as infusions, tea, coffee, and cocoa for both its pleasant flavor and stimulatory effects [23]. Caffeine is arguably the most widely consumed psychoactive and CNS-stimulating compound in the world [24]. Next to its stimulating effects, caffeine has demonstrated antioxidant properties [25]. Other antioxidants were also found in coffee, including polyphenols [26]. The key biologically active polyphenol in coffee is chlorogenic acid which has important antioxidant, anti-inflammatory and antimicrobial properties [27,28]. Antioxidants protect cells from damage by neutralizing free radicals and inhibiting lipid peroxidation [29], promote angiogenesis, improve microcirculation [30], and stimulate cell metabolism [31]. It has been suggested that caffeine may inhibit 5-α-reductase—an enzyme which is a key player in the development of androgenetic alopecia (AGA) [32].

## 3. Caffeine and Adenosine Receptors

**Figure 2:**
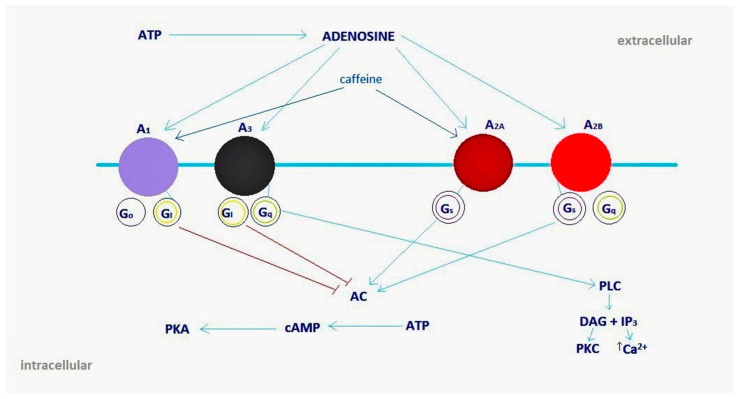
Adenosine receptors as the targets for caffeine. Abbreviations: A_1_ (purple circle), A_3_ (black), A_2A_ (burgundy), A_2B_ (red)—subtypes of adenosine receptors; Go, G_I_, Gs—adenosine receptor-coupled proteins; AC—adenylate cyclase; ATP—adenosine triphosphate; cAMP—cyclic adenosine monophosphate; DAG—diacylglycerol; G—G protein; IP3—inositol triphosphate; PKA—protein kinase A; PKC—protein kinase C; PLC—phospholipase C; caffeine—non-selective adenosine antagonist for A_1_/A_2A_ receptors. Blue arrows symbolize stimulation or conversion; red, blunt-ended lines symbolize inhibition.

Caffeine is structurally related to adenosine and acts as an antagonist of its receptors A_1_ and A_2A_ [15]. This effect is due to the competitive binding of caffeine and paraxanthine to adenosine receptors which have a direct neuromodulatory effect on the CNS. Adenosine is an endogenous neuromodulator with a primarily inhibitory effect; therefore adenosine antagonism by caffeine results in stimulation [32]. By blocking the inhibitory effects of adenosine on its receptors, caffeine indirectly influences the release of dopamine, norepinephrine, acetylcholine, serotonin, glutamate, and gamma-aminobutyric acid (GABA) [33]. An overview of the effects of caffeine on adenosine receptors is shown in Figure 2.

## 4. Metabolic Pathways of Caffeine

**Figure 3:**
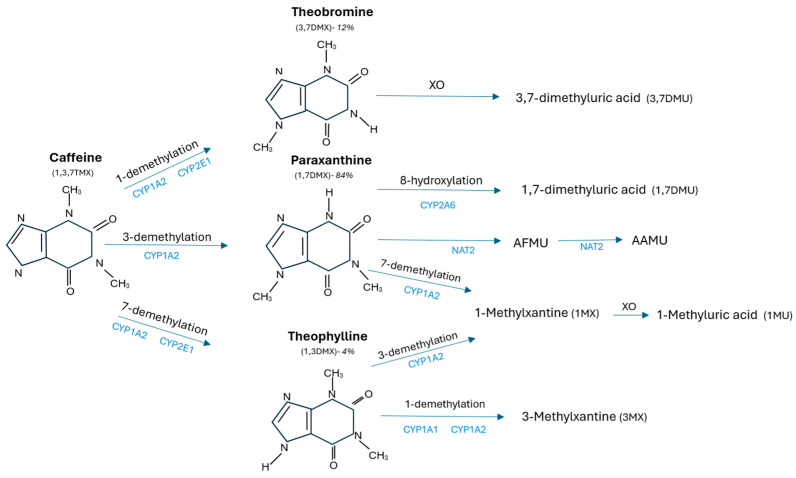
Major metabolic pathways of caffeine. Abbreviations: XO—xanthine oxidase; AFMU—5-acetylamino-6-formylamino-3-methyluracil; AAMU—5-acetamido-6-amino-3-methyluracil.

Caffeine is metabolized in humans into about 25 metabolites [34]. Its metabolism occurs primarily in the liver with the involvement of the cytochrome CYP1A2. The first step in caffeine metabolism is demethylation, which results in the formation of paraxanthine, theobromine, and theophylline (Figure 3). Paraxanthine constitutes 84% of total caffeine metabolites [1]. They are further converted into uric acid which ultimately is excreted in the urine. The enzymes xanthine oxidase and N-acetyltransferase 2 (NAT2) also participate in caffeine metabolism. The main metabolic pathways are demethylation and hydroxylation of the 8-position, which leads to the formation of the corresponding uracil and uric acid derivatives (Table 2) [32].

## 5. Pharmacodynamics of Caffeine in the Skin

The pharmacodynamics of caffeine in the skin include its absorption, penetration into skin cells, and local metabolism. Absorption of caffeine through the skin is limited by its hydrophilic properties [38,39]. Therefore, cosmetic companies are looking for formulations that would increase its penetration [40]. The terms “absorption” and “penetration” are often incorrectly used interchangeably. Penetration is measured by the amount of substance that passes through the skin per area unit and time unit. Absorption, on the other hand, refers to the amount of substance that accumulates in the skin over a given period of time. The accumulated substance may remain in the structures of the skin (epidermis, dermis, and HFs) or penetrate deeper toward blood vessels and further systemic circulation.

**Figure 4:**
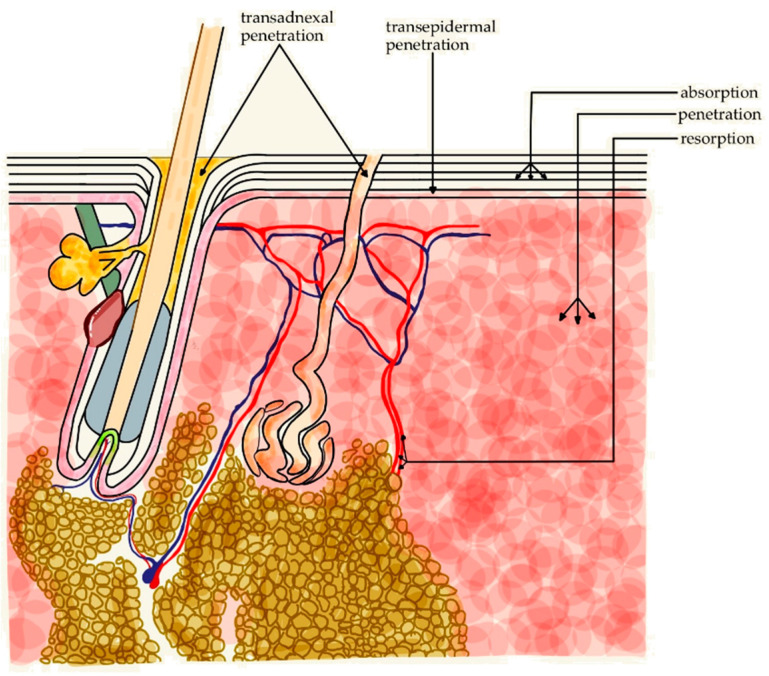
Routes of caffeine penetration into and through the skin.

There are two main routes by which topical preparations can enter the skin [41]:

- Transepidermal (through the stratum corneum);
- Transadnexal (through the skin appendages, i.e., sweat glands and HFs, as shown in Figure 4).

Transadnexal route seems a more efficient way for caffeine to penetrate the skin. While most of the metabolism of caffeine occurs in the liver, the skin also has enzymes that can metabolize it to some extent. As the capacity of the dermal cytochrome P450 system is much lower than in the liver, caffeine remains largely unmetabolized in the skin and interacts with local receptors [42]. Transadnexal transportation via HFs, referred to as transfollicular transportation, has become a subject of intensive research in recent years because the HFs seem to act both as a reservoir for exogenous caffeine and the location of target structures such as stem cells. The accumulation of caffeine in the follicles is 10 times greater than in the surrounding skin [43]. While some substances can penetrate deep into the HF, few are able to penetrate from the follicle into the surrounding skin and microcirculation, which may be advantageous to the case of hair loss products due to accumulation of active molecule in the target structures. An obstacle in understanding these processes is the lack of a suitable model of intact human skin to study transport through the transfollicular route. In cases where HFs are the route, rather than the ultimate target for topical formulations, the penetration from the HF to the surrounding skin may be facilitated by the use of triggered release mechanisms. A number of release triggers are currently being investigated, including radiofrequency, ultrasound, light, enzymatic reactions, and pH manipulation [43].

## 6. The Risk of Adverse Effects of Topical Caffeine

There is an ongoing debate about beneficial or detrimental effects of caffeine which, among other factors, depend on the doses taken [44]. The adverse effects of caffeine consumption are well known and include numerous pharmacological and physiological effects, including influences on the cardiovascular, respiratory, renal, and smooth muscle systems, as well as effects on mood, memory, alertness, and physical and cognitive performance [33]. However, the molecular mechanism responsible for these effects of caffeine are still not fully understood [45]. Untoward health effects of coffee appear to be related to other components of the coffee brew, rather than caffeine itself. These potentially harmful components of coffee include chlorogenic acids, trigonelline, N-methylpyridine, the diterpenes kahweol and cafestol, polysaccharides, peptides, and melanoidins [44]. With regard to caffeine, the Cosmetic Ingredient Review Expert Panel reviewed the safety data and concluded that caffeine used in cosmetic skin products is safe and well tolerated under the current use practices and concentrations [46].

## 7. Caffeine and Stem Cells

Each HF contains a cluster of stem cells residing in an anatomical niche, referred to as the bulge, which is located near the attachment of the arrector pili muscle [47]. These HF stem cells (HFSCs) remain quiescent for most of the hair growth cycle, but in the early anagen phase they proliferate to germ cells and further to matrix cells from which the new hair emerges [48]. The effects of caffeine on skin stem cells, and specifically the HF, have been extensively studied [49]. Caffeine is known for its ability to penetrate the HF and influence hair growth by the anagen (growth) phase. It has been shown to stimulate HF cells, including dermal papilla cells, which play a key role in regulating hair growth [50]. Caffeine achieves this in part by affecting levels of cyclic AMP (cAMP), a signaling molecule that influences cell growth and proliferation, thereby supporting the growth of hair stem cells [51]. The molecular effects of caffeine may increase the proliferation of keratinocytes and dermal papilla cells, which are the key players in maintaining the hair growth cycle [52]. By modulating pathways such as cAMP/PKA (protein kinase A), caffeine promotes cell survival and growth in the HF environment, including HF stem cells [51].

## 8. The Skin from a Molecular Viewpoint

**Figure 5:**
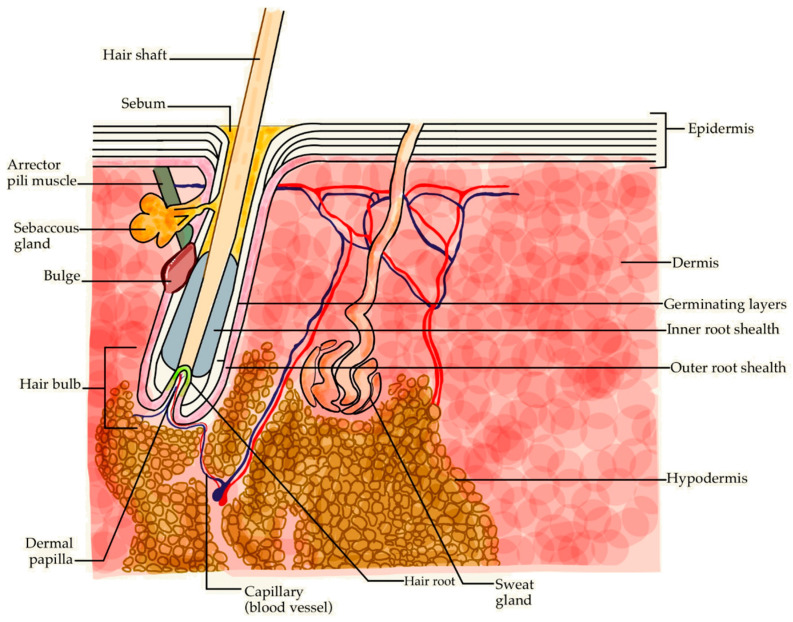
Structure of the skin and hair follicle.

The skin is the largest human organ, with a surface area of approximately 1.5–2 m^2^ and weight of 10–20% of the total body mass [53,54]. Primarily, it is a protective barrier organ that plays a significant role in thermoregulation, homeostasis, sensory perception, and immune defense [55,56,57]. In the embryonic stage, skin development is influenced by molecular cues that control differentiation and maturation of skin structures including HFs. A complex and orchestrated interplay of these pathways is essential for the development and function of healthy skin [58,59]. The skin comprises three primary layers: the epidermis, dermis, and hypodermis with the HF being a sac of invaginated epidermis and dermis (Figure 5).

The epidermis is composed of five distinct layers:

- The basal layer is the deepest layer of proliferating keratinocytes, it also contains Merkel cells that are responsible for tactile sensations [60];
- The stratum spinosum, which is the thickest layer of the epidermis consisting of spiny-shaped keratinocytes, also harbors Langerhans cells, which are the main components of the skin’s immune response [61];
- The granular layer formed of keratinocytes containing keratohyalin granules—formations that mainly consist of keratin, profilaggrin, loricrin and trichohyalin [62]. The granules are also filled with glycolipids that support cell cohesion and contribute to the integrity of the skin within the granular layer [63];
- The clear layer (stratum lucidum) that occurs only on the palms of the hands and the soles of the feet in order to prevent the cracking of the thick skin [64];
- The stratum corneum which is the most superficial layer, consisting of korneocytes—non-viable keratinocytes devoid of nuclei and organelles. Arranged in stratified horny layer of various thickness, stratum corneum acts as a physical skin barrier [65].

The dermis is a fibrous structure consisting of elastic and collagen fibers. It is divided into the superficial papillary layer and the deeper reticular layer [58]. The dermis harbors skin appendages such as sebaceous glands, HFs, sweat glands, as well as sensory neurons, blood vessels, and muscles [66]. Sebaceous glands are exocrine glands, arranged into lobes and ducts and are distributed over the entire body surface except for the hands, feet, and lips. The lobes consist of sebocytes, which produce sebum—a lipid secretion that lubricates and greases the hair and the skin and shows bactericidal and fungicidal properties [67]. Sebaceous glands are an integral part of the pilosebaceous unit (PSU) with several glands emptying into each HF. The sweat glands are divided into eccrine glands dispersed over the entire body except for the lips and genitals, and apocrine glands, which are located in the armpits, around the areola of the breast, around the anus, and on the genitals [67]. The subcutaneous tissue is the deepest layer of the skin that consists mainly of adipose tissue. Its function is to protect the body against temperature changes, as well as harbor HFs, nerves, blood vessels, and muscles [68].

## 9. Caffeine and the Skin

One of the primary roles of the skin is to serve as a physicochemical barrier. It is designed to prevent the penetration of potentially harmful molecules. The ability of a molecule to penetrate through the skin barrier depends on its molecular weight, lipid solubility, and size. As a rule, stratum corneum can be penetrated by molecules that have the molecular weight of less than 500 dalton (Da) [69]. Some researchers believe that larger molecules can also penetrate through the skin but are rapidly metabolized, which makes them physiologically ineffective [70,71]. Although caffeine has a hydrophilic structure, its molecular weight of 194.2 Da would hint on its ability to penetrate the skin barrier relatively easily [72]. For many years, it was believed that the main route of penetration would be the intercellular route [73]. However, recent studies utilizing Raman spectroscopy in a fresh porcine skin model demonstrated that caffeine accumulates at the skin surface and could not permeate through the stratum corneum, meaning that caffeine gets stuck in the outermost layer of dead keratinocytes [74,75]. In a porcine skin model, the major factor enhancing caffeine penetration into the skin was intensive hydration, followed by skin barrier damage due to the combined effect of surfactants and UVA irradiation [76]. Plasma jet technology, high-frequency ultrasound, sonophoresis-propelled caffeinated nanovesicles, self-emulsifying drug delivery system, solid lipid nanoparticles, enriched cerosomes, and niosome-based polymeric needles were tested as future ways to bypass the barrier, based mainly on the porcine skin models [77,78,79,80,81,82,83,84]. However, a study comparing the results of the ex vivo porcine skin model with penetration measurements in humans, showed that the porcine models might demonstrate a higher caffeine penetration than the actual penetration seen in live human skin [75]. A clinical study of 21 Korean women showed that arc-poration, a method of etching micropores in the stratum corneum using high-voltage electric current, enhances caffeine penetration with a subsequent visible cosmetic improvement of the skin [85]. The impassable barrier of the stratum corneum seems a lesser problem with regard to hair products with caffeine, where HFs are the actual target. Higher absorption of caffeine in skin areas with a higher density of HFs strongly suggests that the follicular route greatly contributes to the penetration of caffeine [15,86].

## 10. The Pilosebaceous Unit (PSU)

**Figure 6:**
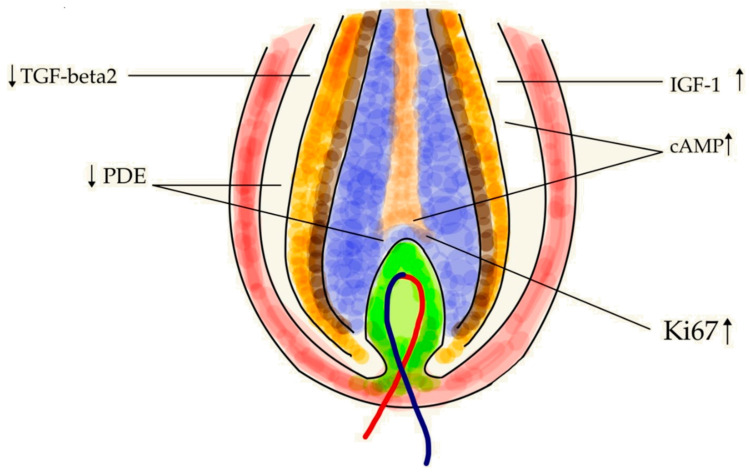
The effects of caffeine in the hair follicle: TGF-beta2—transforming growth factor β2, promotes hair growth [93]; PDE—phosphodiesterase enzyme, regulates cyclic nucleotide signaling and is coupled to diverse physiological functions [94]; IGF-1—insulin-like growth factor 1, initiates and sustains the anagen (growth) phase of the hair cycle [19]; cAMP—cyclic adenosine monophosphate, promotes cell proliferation and metabolic activity in the skin cells and hair follicles [20]; Ki67—antigen Kiel 67, a marker of hair matrix keratinocyte proliferation [51].

The hair follicle, together with the sebaceous glands and the arrector pili muscle, forms the functional entity referred to as the pilosebaceous unit (PSU) [87]. PSUs are the most abundant skin appendages both in terms of body surface area involved and the depth of PSU penetration into the skin. PSU density, size of the follicular openings, follicle volume, and follicle surface area are essential for understanding of the processes determining the penetration of a molecule into the skin via follicle route [15]. PSUs are miniature organs formed during the embryonic period in a complex interaction involving ectodermal and mesodermal structures [88]. They constitute structures essential to facilitating hair growth [89]. The HF consists of a funnel—an epidermal depression evolving into the follicle at the level of sebaceous gland outlets. Between these outlets and the point of arrector pili muscle attachment, a bulge area is located with stem cells. Further down, the growing part of the hair expands at its base to form a bulb seated on a tuft of vascularized, soft connective tissue referred to as the dermal papilla. The hair bulb contains matrix cells that are responsible for the growth of the hair. These regenerative and proliferative areas undergo the cyclical changes in the hair cycle. Histologically, the HF also contains an inner sheath that is divided into the Henle layer, the Huxley layer, the epidermis, and the surrounding outer sheath that contain multipotent stem cells, melanocytes responsible for hair color, and keratinocytes that produce keratin, the main building material of hair. This proliferating part of the hair ends at the level of the of sebaceous glands outlets [66]. Hair follicles and sweat glands were thought to play a secondary role in skin absorption. Their combined share in the total skin surface area is estimated at approximately 0.1% which led to the notion of HFs being rather insignificant in the penetration of substances applied to the skin [90]. These assumptions were, however, based on generalized observations from the forearm skin which it is most frequently used in skin penetration research. It turns out that skin areas with a higher density of follicles, such as the forehead, or with a larger follicular opening, such as the skin in the calf region, show significantly higher penetration; therefore, it is safe to assume a higher transfollicular absorption in such areas [15]. The penetration coefficient through HFs can be several orders of magnitude higher than through the stratum corneum; therefore, the overall contribution of HFs to skin penetration seems relevant [91]. Moreover, the sebum appears to be an excellent penetrating medium which increases the penetration, and thus effectiveness of active substances administered topically [92]. The effects of caffeine in the HF are summarized in Figure 6.

## 11. The Hair and Hair Cycle

The cycle of hair growth and HF regeneration depend on signaling pathways from the surrounding cellular microenvironment including cell-to-cell interactions, hormones, and cytokines [95]. Numerous growth factors, including vascular endothelial growth factor (VEGF), fibroblast growth factor (FGF)-5S, and insulin-like growth factor (IGF)-1, play pivotal roles in the regulation of the HF and hair shaft regeneration. The Wnt/β-catenin signaling pathway is most studied and characterized in this respect; others include bone morphogenetic protein (BMP), Notch, Sonic Hedgehog (Shh), hepatocyte growth factor (HGF), and Eda-A1 [96]. There is increasing evidence that the circadian clock significantly influences hair growth and the hair cycle [97]. The cycle of each HF consists of three cyclically repeating phases: anagen, catagen, and telogen driven by the ability of the HF to produce stem cells [98]. In the early anagen, the hair matrix produces new hair. Anagen lasts between 2 and 6 years. Catagen is the next transitional phase in which the regressive processes in the deepest parts of the HF cause the hair to disconnect from its nourishing blood supply. Catagen lasts one or two weeks and is followed by telogen. In the telogen, the papillary cells separate from the hair shaft, after which they rest for six-weeks, which is followed by the initiation of growth of new hair [2]. During each phase of the hair cycle, the entire hair shaft from tip to root is produced from scratch. Apoptosis is closely correlated with the regression phase by triggering hair follicle cell death and shedding [99]. Cyclic regeneration of the hair shaft and HF is facilitated by HF stem cells—in the catagen and telogen phases, the stem cells from the bulge initiate the next growth phase. The cycle of hair shedding and regeneration continues throughout the entire life span [97].

## 12. Caffeine and the PSU

Caffeine inhibits the enzyme phosphodiesterase which results in an increase in cyclic adenosine monophosphate (cAMP) levels in cells. cAMP stimulates cell metabolism and cell proliferation—a mechanism that may counteract testosterone/dihydrotestosterone-induced miniaturization of the HF in AGA [20,100]. Caffeine also prolongs the duration of the anagen phase by stimulating the proliferation of the hair matrix keratinocytes and increasing the expression of the IGF-1 gene [51]. Caffeine, along with chlorogenic acid—another antioxidant present in coffee—influence hair growth via different pathways, including the upregulation of IGF-1, KGF, and VEGF genes, which leads to the enhanced proliferation of epithelial cells and keratinocytes in the HF, as well as the induction of vasodilation of the surrounding blood vessels [101]. A coffee pulp extract with a high caffeine (1.9% dry weight), flavonoid (0.7%), and phenolic content (0.6%) showed high antioxidant activity and increased cell viability and migration of human hair follicle dermal papilla cells in an in vitro experiment [102].

Caffeine penetrates well into human skin which made it a model substance for skin barrier penetration studies [103,104]. As a result, caffeine has been extensively studied and proven to have a good penetration and good bioavailability after topical application on the scalp [94], which is why it may be found in many products such as shampoos, lotions, liquids, serums, foams, etc. [105]. It has been demonstrated that caffeine penetrates via the HFs faster than via intercellular epidermal route—in fact, HFs are the only route for fast caffeine absorption in the first 20 min after topical application [50]. A study conducted on the legs of nine men showed that a combination of caffeine and piroctone olamine in a shampoo is more effective than a shampoo with caffeine only which suggests that piroctone olamine enhances the penetration of caffeine or its effectiveness in target cells [106]. Both in vivo and ex vivo studies seem to support the conviction of good caffeine penetration into the hair follicles. Nevertheless, the physicochemical properties of caffeine which determine its easy formation of adducts deserve some consideration as a factor that could potentially influence its absorption and penetration into skin structures. Caffeine has been typically used in cosmetic preparations like shampoos, foams, or serums which may consist of several dozen ingredients, including ones that easily form bonds with caffeine. Caffeine increases the hydrophobicity of the water phase in emulsions which allows for the greater integration of oil molecules into the core of water-phase droplets, ultimately resulting in a phase inversion of the emulsion from oil-in-water (O/W) to water-in-oil (W/O) [107].

The solubility of caffeine is mostly affected by polarity or hydrogen bonding. In total, hydrated caffeine forms three hydrogen bonds with its neighboring water molecules [108,109]. Caffeine molecules readily form hydrates, with the first hydration shells containing on average 56 water molecules [108]. Nuclear magnetic resonance (NMR) and a neutron total scattering study demonstrated that caffeine’s water solubility is enhanced by hydrogen bond interactions between water and sodium benzoate, a frequent ingredient of shampoos [110]. Also, citric acid, another ingredient of shampoos, interacts with caffeine molecules through hydrogen bonds [111]. The skin conditioning ingredient, sinapic acid, interacts with caffeine through van der Waals forces and hydrogen bonds, while the antimicrobial and antioxidant ferulic acid spontaneously creates adducts with caffeine via hydrogen bonds [112]. Altogether, this demonstrates that in complex products, other ingredients could greatly influence the bioavailability of caffeine in both positive and negative ways. In products like shampoos, hair tonics, foams, or cosmetic serums, their complexity may require advanced methods of artificial intelligence and machine learning to accurately predict all the possible interactions between caffeine and the remaining ingredients [113,114].

## 13. Caffeine in Hair Loss Products

Caffeine occurs in numerous leave-on cosmetic preparations such as foams [115], serums [116,117], liquids [118], or lotions [119], as well as the rinse-off product shampoo [93,119,120,121]. Its effectiveness in cosmetics for hair loss is subject to intensive research [106]. HFs are an effective entry path for xenobiotics (medicines, toxins, or cosmetic ingredients), because they are surrounded by a dense network of capillaries, dendritic cells, and stem cells [122,123]. Unlike the stratum corneum, which sheds one layer of corneocytes every day and is exposed to repeated washing, rinsing, and wiping, HFs can be viewed as an entry route and long-term reservoir for topically applied substances [124]. According to current knowledge, the follicular reservoir on the scalp is 3–5 times larger than on the forehead and calf regions, making the HF of the scalp a perfect target for topical formulas, including those with caffeine [15]. Available data suggest that caffeine penetrates human HFs within minutes and can remain there for up to 48 h, even after repeated washing, which makes HFs a perfect target for topical treatment with caffeine [104]. This seems to be supported by the results from clinical studies of caffeine-based hair loss products with 67% to 100% participants expressing their satisfaction with the treatments [118,119]. Unfortunately, the majority of clinical trials published so far seem to offer a rather low level of evidence due to considerable flaws in their study design. The major methodological problems include a lack of a control group and an adequate comparator (placebo), lack of information on caffeine concentration in the product tested, or trials of complex products in which caffeine is part of a mixture with other ingredients (Table 3).

## 14. Conclusions

Caffeine is a molecule with multifaceted effects observed at a molecular, cellular, and clinical level that seems very interesting in the context of its possible use in hair loss. At a molecular level, caffeine interacts with the adenosine pathway leading to increased cAMP levels, but it also acts as an antioxidant. The molecule’s ability to accumulate in hair follicles further suggests that it may be a good treatment option against hair loss. Clinical studies, although burdened with methodological flaws, also hint at the effectiveness of caffeine which places the molecule among very promising hair loss remedies.

## 15. Future Directions 

Future research should focus on optimizing the formulation of cosmetic products containing caffeine to ensure its optimal diffusion in the sebum that fills hair follicles. In this way, higher concentrations and thus a better effectiveness of caffeine could be achieved in the target structure. Future, well-designed clinical trials of topical caffeine products against hair loss are needed that are designed in a double-blind, randomized manner and controlled by the use of a suitable comparator (placebo), e.g., an identical hair product devoid only of caffeine, or caffeine being replaced by minoxidil which is well-established therapeutic option in hair loss trials. Such trials should include patients with well-defined clinical diagnoses in groups large enough to produce statistically meaningful data. Taking into account what is already known from the literature about the physicochemical properties and metabolic effects of caffeine, combined with experimental and clinical data, it seems that caffeine could indeed prove to be an effective and safe option in the management of hair loss.

## Figures and Tables

**Table 1:** PubChem characteristic of caffeine [8].

Chemical Name	IUPAC Name	CAS	EC
Caffeine	1,3,7-trimethylpurine-2,6-dione	58-08-2	200-362-1

Abbreviations: IUPAC—International Union of Pure and Applied Chemistry; CAS—Chemical Abstracts Service number; EC—European Community number.

**Table 2:** Biologically active caffeine metabolites.

Chemical Name	IUPAC Name	CAS	EC	Ref.
Paraxanthine	1,7-dimethyl-3*H*-purine-2,6-dione	611-59-6	210-271-9	[35]
Theobromine	3,7-dimethylpurine-2,6-dione	83-67-0	201-494-2	[36]
Theophylline	1,3-dimethyl-7*H*-purine-2,6-dione	58-55-9	200-385-7	[37]

Abbreviations: IUPAC—International Union of Pure and Applied Chemistry; CAS—Chemical Abstracts Service number; EC—European Community number.

**Table 3:** Chronology of clinical studies of caffeine in hair loss.

Year	Study Group	Caffeine Formulation	Comparator	Main Outcome	Evidence Level *	Ref.
2010	30 M with AGA	Shampoo with caffeine	None	Objective and subjective improvement	Very low	[119]
2013	66 M with AGA	Shampoo with caffeine	Shampoo base	Objective and subjective improvement	Very low	[120]
2013	30 F with TE	Shampoo with caffeine	None	Objective and subjective improvement	Very low	[121]
2013	60 M with AGA	Minoxidil and caffeine solution	Minoxidil solution	Caffeine + minoxidil more effective than minoxidil alone	Impossible to assess (article in Persian)	[125]
2017	210 M with AGA	Caffeine solution (0.2%)	Minoxidil solution (5%)	Effects of 0.2% caffeine comparable to 5% minoxidil	Medium	[118]
2022	62 M with AGA	Foam with 10 ingredients including caffeine	Foam base	Foam with caffeine. better than foam base	Medium	[115]
2023	150 M and F with AGA	Serum with a complex mixture including caffeine	None	Improvement of hair parameters in some areas	Very low	[117]
2024	32 F and M with hair loss	Serum with 30 ingredients including caffeine	None	Improvement of hair condition and growth	Very low	[116]
2024	84 F and M with thinning hair	Shampoo with caffeine (0.4%) and adenosine	Shampoo base	Improvement after active shampoo but not the control	Medium	[93]
2024	20 M with AGA	Topical formulation containing caffeine, 3% Procapil™ (Croda International, Snaith, UK) and zinc PCA	None	Improvement in hair growth, decrease in hair loss	Very low	[126]

* Evidence level assessed in line with the GRADE methodology [127]. Abbreviations: AGA—androgenic alopecia; F—females; M—males; TE—telogen effluvium; zinc PCA—zinc pyroglutamate.

## Data Availability

Data are contained within the article.
